# Gamma-Muricholic Acid Inhibits Nonalcoholic Steatohepatitis: Abolishment of Steatosis-Dependent Peroxidative Impairment by FXR/SHP/LXRα/FASN Signaling

**DOI:** 10.3390/nu15051255

**Published:** 2023-03-02

**Authors:** Yang Xie, Feng Shen, Yafang He, Canjie Guo, Ruixu Yang, Haixia Cao, Qin Pan, Jiangao Fan

**Affiliations:** 1 Department of Gastroenterology, Xinhua Hospital, School of Medicine, Shanghai Jiao Tong University, Shanghai 200092, China; 2 Endoscopy Center, Xinhua Hospital, School of Medicine, Shanghai Jiao Tong University, Shanghai 200092, China; 3 Department of Pediatric Respiratory, Xinhua Hospital, School of Medicine, Shanghai Jiao Tong University, Shanghai 200092, China; 4 Department of Gastroenterology, Renji Hospital, School of Medicine, Shanghai Jiao Tong University, Shanghai 200080, China; 5 Research Center, Shanghai University of Medicine & Health Sciences Affiliated Zhoupu Hospital, Shanghai 201318, China; 6 Shanghai Key Lab of Pediatric Gastroenterology and Nutrition, Shanghai 200092, China

**Keywords:** nonalcoholic steatohepatitis, muricholic acid, farnesoid X receptor, steatosis, lipid peroxidation, apoptosis

## Abstract

Nonalcoholic steatohepatitis (NASH) reflects the outcome of steatosis-based peroxidative impairment. Here, the effect and mechanism of γ-muricholic acid (γ-MCA) on NASH were investigated on the basis of its actions in hepatic steatosis, lipid peroxidation, peroxidative injury, hepatocyte apoptosis, and its NAFLD activity score (NAS). The agonist action of γ-MCA on farnesoid X receptor (FXR) upregulated the small heterodimer partner (SHP) expression of hepatocytes. An increase in SHP attenuated the triglyceride-dominated hepatic steatosis which was induced in vivo by a high-fat high-cholesterol (HFHC) diet and in vitro by free fatty acids depending on the inhibition of liver X receptor α (LXRα) and fatty acid synthase (FASN). In contrast, FXR knockdown abrogated the γ-MCA-dependent lipogenic inactivation. When compared to their excessive production in HFHC diet-induced rodent NASH, products of lipid peroxidation (MDA and 4-HNE) exhibited significant reductions upon γ-MCA treatment. Moreover, the decreased levels of serum alanine aminotransferases and aspartate aminotransferases demonstrated an improvement in the peroxidative injury of hepatocytes. By TUNEL assay, injurious amelioration protected the γ-MCA-treated mice against hepatic apoptosis. The abolishment of apoptosis prevented lobular inflammation, which downregulated the incidence of NASH by lowering NAS. Collectively, γ-MCA inhibits steatosis-induced peroxidative injury to ameliorate NASH by targeting FXR/SHP/LXRα/FASN signaling.

## 1. Introduction

Nonalcoholic fatty liver disease (NAFLD) is liver-specific metabolic stress characterized by hepatic steatosis, with a spectrum encompassing nonalcoholic fatty liver (NAFL), nonalcoholic steatohepatitis (NASH), liver fibrosis/cirrhosis, and hepatocellular carcinoma [1]. The prevalence of NAFLD has risen to around 25% worldwide, reflecting major chronic liver disease [2]. NASH, instead of NAFL, takes the critical step toward NAFLD-related disability and mortality [3].

The onset of NASH undergoes a lot of interactive mechanisms, such as obesity, genetic predisposition, epigenetic regulation, and gut dysbiosis [4]. Microbial metabolites underlie, to a large extent, the effect of gut dysbiosis on NASH [5]. By their role in intestinal lipid absorption and their stimulatory impact on bile acid receptors (e.g., FXR, Takeda G protein-coupled receptor 5 (TGR5), pregnane X receptor), bile acids have been demonstrated to regulate host lipid metabolism [6]. Moreover, abnormalities in the bile acid pool and its composition (e.g., conjugated cholic acid) exhibit a close association with mammalian NASH, mainly based on lipid accumulation, hepatocyte injury, and related inflammation [7,8].

Being derived from α-muricholic acid (α-MCA) or β-muricholic acid (β-MCA), γ-muricholic acid (γ-MCA) reflects one kind of low-level secondary bile acid in the bile acid pool of mice [9]. It shares the molecular formula, 3,6,7-trihydroxy-5β-cholan-24-oic acid, with α-MCA and β-MCA that dominate rodent MCAs. When compared to 3α, 6β, 7α-trihydrol in α-MCA, and 3α, 6β, 7β-trihydrol in β-MCA, the 3α, 6α, 7α-trihydrol in γ-MCA confers a hydrophobicity-dependent affinity to farnesoid X receptor (FXR) [10,11]. Furthermore, the poor binding capacity of α-MCA/β-MCA and the hydrophobic pocket of FXR qualifies them as FXR-specific antagonists [12]. In contrast, the stereostructure of the 6α hydroxyl group in γ-MCA may restore the activity of FXR against α-MCA/β-MCA by its pocket-occupying capability [13]. Given the inhibitory roles of FXR in hepatic lipogenesis and the lobular inflammation upon steatosis-related lipid peroxidation, γ-MCA is suggested to serve as a therapeutic agent of NASH.

Therefore, we established experimental NASH in mice by a high-fat high-cholesterol (HFHC) diet and performed 10 mg/kg or 100 mg/kg γ-MCA daily administration for 16 weeks. Both oil red O staining and triglyceride (TG) analysis revealed the effect of γ-MCA on hepatic steatosis. Then, liver concentrations of malondialdehyde (MDA) and 4-hydroxynonenal (4-HNE) exhibited steatosis-dependent lipid peroxidation. The lipid peroxidative injury and related hepatocyte apoptosis were further investigated by transferase levels and a terminal deoxynucleotidyl transferase-mediated dUTP nick end labeling (TUNEL) assay, respectively. Pathological evaluation and the NAFLD activity score (NAS) finally demonstrated the outcome of γ-MCA treatment. Mechanically, γ-MCA-based activation of FXR and downstream signaling of small heterodimer partner (SHP)/liver X receptor α (LXRα)/fatty acid synthase (FASN) was analyzed in vivo and in vitro.

## 2. Materials and Methods

### 2.1. Establishment of Hepatocellular Steatosis and γ-MCA Administration

Alpha mouse liver 12 (AML12) cells (Cell Bank of Chinese Academy of Sciences, Shanghai, China) were cultured with DMEM/F12, 1% ITS Liquid Media Supplement, 40 ng/mL dexamethasone, 10% fetal bovine serum (FBS), and 1% Penicillin-Streptomycin Solution. They were divided into groups of normal control (NC), free fatty acid (FFA), FFA + 10 μM γ-MCA, and FFA + 100 μM γ-MCA (3 wells per group) at random. With an exception of the NC group, AML12 cells in each group were subjected to the induction of hepatocellular steatosis using 1.5 mM oleate/palmitate (oleate:palmitate = 2:1) for 24 h [14]. The other 2 groups were simultaneously administrated by 10 μM and 100 μM γ-MCA, respectively, during the same period.

### 2.2. Induction of Rodent NASH and γ-MCA Treatment

A total of 40 adult male specific pathogen-free (SPF) c57BL/6 mice (Jihui Laboratory Animal Care Co., Shanghai, China) were randomized into the normal control (NC), NASH, NASH + vehicle, NASH + 10 mg/kg γ-MCA, and NASH + 100 mg/kg γ-MCA groups (8 mice per group), respectively. Except for those in the NC group with a normal diet, all the mice were exposed to the HFHC diet (2% cholesterol, 10% lard, and 88% normal diet) for 16 weeks [15]. Simultaneously, mice in the NASH + 10 mg/kg γ-MCA and NASH + 100 mg/kg γ-MCA groups were daily intragastrically administrated with 10 mg/kg and 100 mg/kg γ-MCA (H915264, Macklin, Shanghai, China), respectively, in 200 μL water for 16 weeks. The liver index of the mice was calculated as follow: liver index = liver weight / body weight. The sample size was estimated by the prospective difference in the steatotic incidence between the NASH and NASH + 100 mg/kg γ-MCA groups using Gpower v3.1.9.7 software [16]. The animal experiments were conducted according to the Guide for the Care and Use of Laboratory Animals (1996), with the approval of the ethical committee of Xinhua hospital.

### 2.3. Pathological Assessment

Mice from each group were sacrificed to collect liver samples at the end of the 16th week. H and E staining was performed on each sample to evaluate the pathological indexes associated with NASH, including hepatocyte steatosis, ballooning degeneration, and lobular inflammation. A NAFLD activity score (NAS) was finally employed to diagnose NAFL, borderline-nonalcoholic steatohepatitis (NASH), and NASH [17]. Pathological diagnosis of each sample was performed in a blinded manner by a pathologist with diagnostic experience (Q.P.).

### 2.4. Assay for Serum Transferases

Serum samples of each group were obtained from the blood by centrifugation at 3000 rpm and 4 °C for 30 min. The serum levels of alanine aminotransferase (ALT) and aspartate aminotransferase (AST) were assessed using an ALT Assay Kit (C009-2-1, Jiancheng, Nanjing, China) and an AST Assay Kit (C010-2-1, Jiancheng), respectively, according to the same protocol as follows. The reagent 1 was warmed using water bath at 37 °C, incubated with 20 μL serum samples at 37 °C for 30 min, and mixed with 20 μL 2,4-dinitrophenylhydrazine at 37 °C for 20 min, subsequently. Then, the mixture was subjected to chromogenic reaction with 200 μL 0.4 mol/L NaOH at room temperature for 15 min, and OD analysis at 510 nm using the microplate spectrophotometer (Epoch, Agilent, Santa Clara, CA, USA). The serum levels of ALT and AST were finally calculated according to the multinomial standard curves, which were established using sodium pyruvate via gradient concentration.

### 2.5. Oil Red O (ORO) Staining

To test the existence and localization of neutral lipids, ORO staining was carried out in both AML12 cells and frozen sections of liver samples. The ORO working solution was prepared using saturated ORO solution diluted in water to 60% of the volume fraction and heated at 65 °C for 30 min. The AML12 cells were washed using phosphate buffer saline (PBS), fixed using 4% paraformaldehyde for 10 min, washed again, and treated with 60% isopropanol for 15 s. The frozen sections were warmed at room temperature for 10 min. Next, the cells and frozen sections were stained using ORO working solution in the dark for 30 min and 10 min, respectively, treated with 60% isopropanol for 5 s, and washed in water for 15 min. Finally, the cells and frozen sections were counterstained using hematoxylin for 3 min, differentiated using 1% hydrochloric acid alcohol for 20 s, and neutralized using 3% ammonia water for 5 s.

### 2.6. Triglyceride (TG) Analysis

The liver tissue of each mouse was ground into homogenate and centrifuged to obtain the supernatant. The total protein concentration of the supernatant was determined using a BCA protein concentration determination kit G2026 (Servicebio, Wuhan, China) according to the manufacturer’s instructions. The TG content in liver samples was then measured using the TG Determination Kit (GPO-PAP method) (C061, Huili) against the total protein concentration [18]. In detail, a 250 μL GPO-PAP working solution was mixed with 2.5 μL PBS, standard glycerol (1.7 mmol/L), or supernatant. The mixture was subjected to OD analysis at 510 nm using the automatic biochemistry analyzer. The TG concentration was resultantly determined using the colorimetric method with standard glycerol normalized by PBS.

### 2.7. Assays for Lipid Peroxidation

To evaluate the indexes of lipid peroxidation, the concentration of MDA was determined in the supernatant of liver samples by the thiobarbituric acid method using the MDA Assay Kit (A003-1, Jiancheng, Nanjing, China). The working solution of the MDA Assay Kit was mixed with 200 μL supernatant, 10 nmol/mL standard, and ethanol. The mixture was heated at 95 °C for 40 min and centrifuged for supernatant at 4000 rpm for 10 min. Being normalized by that of ethanol, the OD of the supernatant at 532 nm colorimetrically reflected the MDA concentration.

The hepatic 4-HNE concentration was also investigated by the enzyme-linked immunosorbent assay (ELISA) method using a Mouse 4-HNE Assay Kit (F15653, Westang, Shanghai, China). In succession, 5 μL of the supernatant of liver samples was mixed with 100 μL 4-aminoantipyrine solution in the coated wells, incubated with 20 μL enzyme-conjugated antibody at 37 °C for 15 min, and subjected to the DENLEY DRAGON Wellscan MK 3 (Thermo Fisher Scientific, Waltham, MA, USA) at 550 nm. The 4-HNE concentration was finally calculated according to the standard curve. Both MDA and 4-HNE contents were calculated using MDA and 4-HNE concentrations against the total protein concentration, respectively.

### 2.8. TUNEL Assay

Sections of liver samples were exposed to deparaffinization, incubation with 20 μL/mL proteinase K at 37 °C for 20 min, permeabilization at room temperature for 20 min, and blockage with 3% hydrogen peroxide buffer at room temperature for 20 min. Afterwards, the TUNEL Cell Apoptosis Detection Kit (G1507, Servicebio, China) was employed to highlight the apoptotic cells in the liver. Briefly, the TdT incubation buffer was prepared using recombinant TdT enzyme, biotin-dUTP labeling mix, and equilibration buffer in 1:5:50 ratios of volume. Liver sections were then incubated with 50 μL equilibrium buffer at room temperature for 10 min, and 56 μL TdT incubation buffer in a wet box at 37 °C for 1 h. After reaction with 100 μL streptavidin-HRP buffer at 37 °C for 30 min, sections were incubated with 100 μL 3,3′-diaminobenzidine (DAB) (G1212, Servicebio) working solution, microscopically observed for positive staining, and washed using water to terminate the chromogenic reaction. Pictures from 20× scopes of sections were collected and counted using ImageJ software (https://imagej.nih.gov/ij/, accessed on 8 November 2022) for the number of positive cells in every 20× scope. The positive cells were identified using the “Color Threshold” function, with no color pass of blue and an appropriate brightness threshold that excluded normal cells and covered positive cells. After the transformation of the image type into 8-bit, the “Fill Holes” and “Watershed” functions were applied to fill the holes within every positive cell and separate the overlapping positive cells. The numbers of positive cells were counted using the “Analyze Particles” function automatically, with the unit of numbers per 20× scope.

### 2.9. RNA Interference

Small interfering RNA (siRNA) targeting mouse FXR (siRNA-FXR) and its negative control (siRNA-NC) were synthesized by GenePharma (Shanghai, China). The sequences of siRNA-FXR were 5′-CAGGUUUGUUAACUGAAAUTT-3′ (sense) and 5′-AUUUCAGUUAACAAACCUGTT-3′ (antisense). Being randomized into siRNA-FXR and siRNA-NC groups, the AML12 cells were transfected with siRNA-FXR or siRNA-NC using LIPOFECTAMINE 3000 (L3000015, Thermo Fisher Scientific) according to the instructions of the manufacturer. The effect of RNA interference on FXR was tested by reverse transcription-quantitative polymerase chain reaction (RT-QPCR) and Western blot.

### 2.10. RT-QPCR

The total RNA of the liver samples was extracted using RNAiso Plus (9109, Takara Bio, Shiga, Japan) and subjected to reverse transcription using the PrimeScript™ RT Reagent Kit (RR036, Takara Bio) according to the manufacturer’s instructions. Thereafter, quantitative PCR was performed using the Hieff UNICON^®^ qPCR SYBR^®^ Green Master Mix (Low Rox) (11199ES, Yeasen Biotechnology, Shanghai, China) on QuantStudio 3 (Thermo Fisher Scientific, Waltham, MA, USA) with three replications. Relative expression levels were normalized to the glyceraldehyde-3-phosphate dehydrogenase (GAPDH) housekeeping gene using the 2^-ΔΔct^ method. The primers used in the experiments are shown in Table 1.

### 2.11. Western Blot

Protein samples were extracted from mouse liver using RIPA lysis buffer and quantified by the BCA Protein Assay Kit (Beyotime, Shanghai, China). Total protein extracts were incubated with loading buffer at 100 °C for 10 min and subjected to electrophoresis on 8% or 10% sodium dodecyl sulfate-polyacrylamide gel. Afterward, proteins were transferred onto polyvinylidene difluoride membranes. The membranes were incubated with protein-free rapid blocking buffer and subsequently with primary antibodies against α-tubulin (1:1000, 66031, Proteintech, Rosemont, IL, USA), FXR (1:1000, 25055, Proteintech), SHP (1:500, sc-271511, Santa Cruz, Dallas, TX, USA), LXR (1:2000, 14351, Proteintech), and FASN (1:1000, 10624, Proteintech) overnight at 4 °C, and finally with horseradish peroxidase (HRP)-conjugated secondary antibodies at room temperature for 1 h. Protein bands were visualized by chemiluminescence using Amersham Imager 680 (AI680, Cytiva, Tokyo, Japan) and assessed using ImageJ software.

### 2.12. Immunohistochemistry

Sections of liver samples were subjected to antigen retrieval using citrate buffer at 95 °C for 3 min. The blockage was performed using 3% hydrogen peroxide buffer in the dark for 25 min and 3% bovine serum albumin for 30 min at room temperature. Then, the liver sections were reacted with primary antibodies specific to SHP (1:50, sc-271511, Santa Cruz), LXR (1:100, 14351, Proteintech) and FASN (1:100, 10624, Proteintech), respectively, in a wet box overnight at 4 °C. After labeling with HRP-conjugated secondary antibodies (A0208 and A0216, Beyotime, Shanghai, China), immunohistochemistry signals were visualized using the DAB method. The semiquantitative analyses were performed using ImageJ software. After the exclusion of normal cell nuclei using the “Color Threshold” function and the transformation of the image type into 8-bit, the positive part of each picture was identified using the auto “Threshold” function and subjected to integral calculus for the average optical density (OD) according to the following formula (OD(x,y) ds, OD of each pixel in the positive staining area, and S, total positive staining area):

$$\mathrm{Average}\;\mathrm{OD}=\int_{S}^{}\mathrm{OD}\left(x,y\right)\;\mathrm{ds}/\;S$$

### 2.13. Statistical Analysis

The numerical variables of this study were presented as the mean ± standard error (SEM). The Kolmogorov–Smirnov test and the Brown–Forsythe test were applied for assessing the normality and homogeneity of the variance of the data, respectively. One-way ANOVA was performed to investigate the difference between multiple groups, while the difference between the two groups was analyzed using Tukey’s multiple comparisons test for the data with a normal distribution and Dunn’s multiple comparisons test for the ranked data. Correlation analyses were conducted using the Pearson correlation test between two data with a normal distribution or the Spearman correlation test between normal-distributed data and ranked data. In addition, a chi-square test was performed to investigate the difference in pathological classification and was adjusted using Bonferroni correction for multiple comparisons between two groups. SPSS version 23.0 (SPSS Inc., Chicago, IL, USA) was used for the statistical analyses. A two-side significance threshold was set at *p* < 0.05.

## 3. Results

### 3.1. γ-MCA Targeted FXR/SHP/LXRα/FASN Signaling to Inhibit High Fat-Stimulated Hepatocellular Steatosis

**Figure 1:**
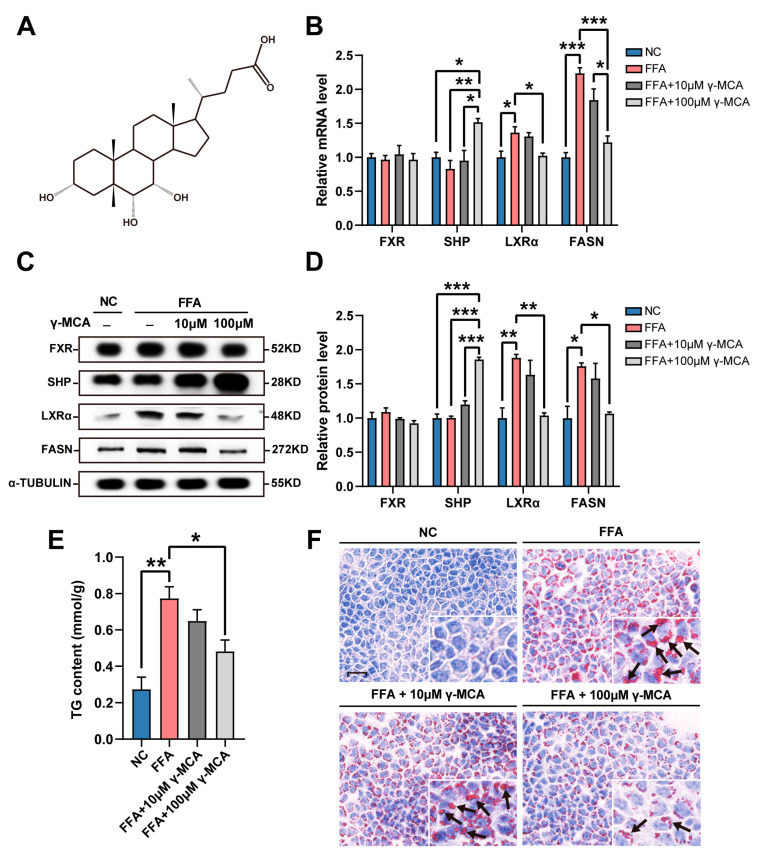
γ-MCA alleviated hepatocellular steatosis by the activation of FXR/SHP/LXRα/FASN signaling. (**A**,**B**) γ-MCA (**A**) affected AML12 cells, especially at the concentration of 100 μM, to activate the transcription of SHP and restore the FFA-stimulated abnormalities in LXRα and FASN mRNAs (**B**). (**C**,**D**) γ-MCA administration increased the SHP expression of AML12 cells, whereas it downregulated their LXRα and FASN levels (**C**) in a dose-dependent manner (**D**). (**E**) FFA stimuli led to TG accumulation, which was attenuated by 100 μM γ-MCA. (**F**) Oil Red O staining exhibited an augmentation in the number of neutral lipid droplets in the FFA group, and a decrease in the FFA + 100 μM γ-MCA group (scale bar: 25 μm). Arrows indicate the lipid droplets in the cytoplasm of hepatocytes. The data were presented as the mean ± SEM. *, *p* < 0.05; **, *p* < 0.01; and ***, *p* < 0.001. free fatty acid, FFA; muricholic acid, MCA; normal control, NC; farnesoid X receptor, FXR; small heterodimer partner, SHP; liver X receptor α, LXRα; fatty acid synthase, FASN.

When compared to those in the NC group, AML12 cells without γ-MCA administration (FFA group) exhibited a significant increase in LXRα and FASN levels after the FFA stimuli (Figure 1A,B). In the groups of FFA + 10 μM γ-MCA and FFA + 100 μM γ-MCA, an agonist effect of γ-MCA on FXR upregulated SHP expression at both the transcriptional and translational levels in a dose-dependent manner (Figure 1B–D). The SHP-induced LXRα inhibition subsequently abolished its transcription-activating role of FASN (Figure 1B–D). In contrast to their upregulation in the FFA group, lipid droplets and TG content were resultantly decreased by γ-MCA administration, especially in the FFA + 100 μM γ-MCA group (Figure 1E,F).

**Figure 2:**
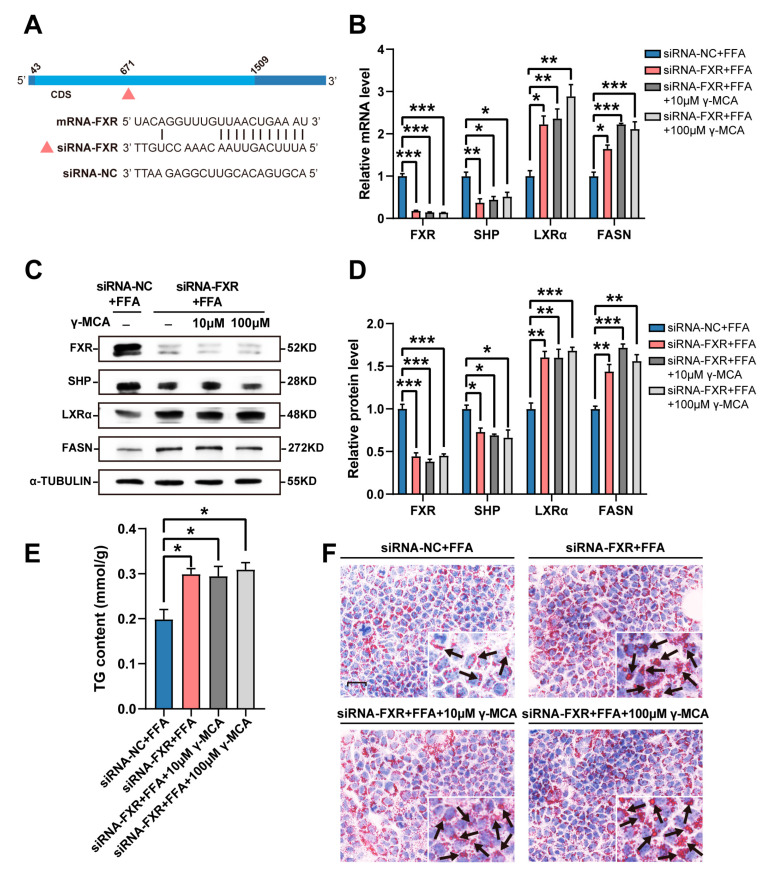
FXR deficiency abolished the inhibitory effect of γ-MCA on hepatocellular steatosis. (**A**) siRNA-FXR, but not the negative control (siRNA-NC), targeted FXR mRNA by base complementation. (**B**) siRNA-FXR degraded FXR mRNA and then transcriptionally deprived the activation of FXR/SHP/LXRα/FASN signaling upon γ-MCA administration. (**C**,**D**) Western blot (**C**) and quantitative analysis (**D**) exhibited the inactivation of γ-MCA-induced SHP, LXRα, and FASN expression upon the condition of FXR knockdown. (**E**,**F**) FXR knockdown abolished the γ-MCA-dependent downregulation of TG concentration (**E**) and hepatocellular steatosis (scale bar: 25 μm) (**F**). Arrows indicate the lipid droplets in the cytoplasm of the hepatocytes. The data were presented as the mean ± SEM. *, *p* < 0.05; **, *p* < 0.01; and ***, *p* < 0.001.

Furthermore, FXR-specific RNA interference was carried out to confirm the target effect of γ-MCA (Figure 2A). FXR knockdown fundamentally abrogated the γ-MCA-dependent SHP upregulation and then rescued the SHP-based inhibition of LXRα and FASN, with a significant loss of SHP expression and gain of LXRα and FASN expression compared to the negative control of RNA interference (Figure 2B–D). As a result, γ-MCA failed to ameliorate the high fat-stimulated hepatocellular steatosis at concentrations from 10–100 μM, with a higher TG level in the FXR knockdown than the negative control (Figure 2E,F). γ-MCA, therefore, prevented hepatic lipogenesis by targeting FXR/SHP/LXRα/FASN signaling.

### 3.2. γ-MCA Attenuated Rodent Liver Steatosis Induced by the HFHC Diet

**Figure 3:**
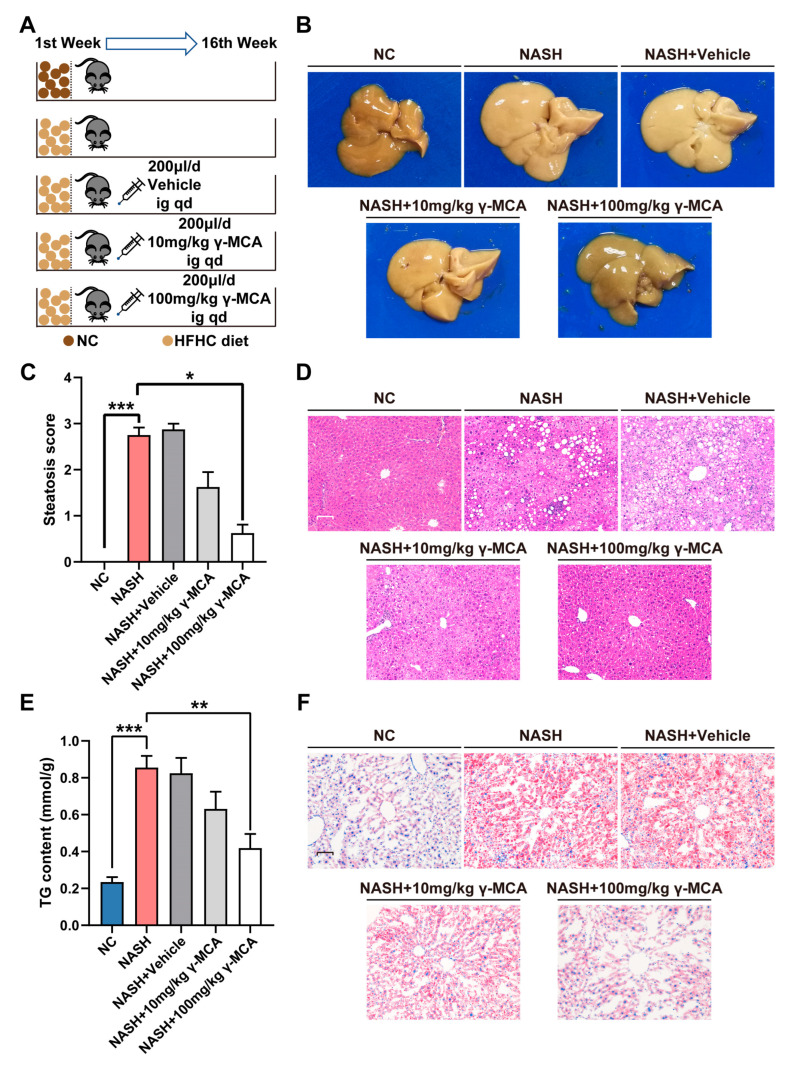
γ-MCA relieved the HFHC diet-induced hepatic steatosis in mice. (**A**) Except for those in the NC group with a normal diet, mice in the NAFLD, NAFLD + Vehicle, NAFLD + 10 mg/kg γ-MCA, and NAFLD + 100 mg/kg γ-MCA groups were exposed to a high-fat high-cholesterol (HFHC) diet for 16 weeks. When compared to the intragastric administration of vehicle (200 μL/day) in the NAFLD + Vehicle group, the NAFLD + 10 mg/kg γ-MCA and NAFLD + 100 mg/kg γ-MCA groups were treated by gavage of 10 mg/kg and 100 mg/kg γ-MCA in vehicle, respectively. (**B**) An enlarged liver, with a blunted edge and yellow-in-color, featured the gross appearance of the NAFLD group, whereas improvements were shown in the NAFLD + 10 mg/kg γ-MCA and NAFLD + 100 mg/kg γ-MCA groups. (**C**,**D**) Both steatosis score (**C**) and H and E staining (**D**) demonstrated the occurrence of hepatic steatosis in the NAFLD group and its resolution in the NAFLD + 10 mg/kg γ-MCA and NAFLD + 100 mg/kg γ-MCA groups (scale bar: 50 μm). (**E**,**F**) Both TG analysis (**E**) and Oil Red O staining (**F**) characterized the lipid accumulation in the NAFLD group, and the lipolysis in NAFLD + 10 mg/kg γ-MCA and NAFLD + 100 mg/kg γ-MCA groups (scale bar: 50 μm). The data were presented as the mean ± SEM. *, *p* < 0.05; **, *p* < 0.01; and ***, *p* < 0.001.

After administration of the HFHC-diet for 16 weeks (Figure 3A), liver steatosis was established in the NASH group with characteristics of a beige appearance (Figure 3B), hepatocyte steatosis (Figure 3C,D), and TG accumulation (NC vs. NASH: 0.2344 ± 0.0265 mmol/g vs. 0.8556 ± 0.0630 mmol/g, *p* < 0.0001) (Figure 3E,F). Contrastively, mice in both NASH + 10 mg/kg γ-MCA and NASH + 100 mg/kg γ-MCA displayed improvement in their gross appearance (Figure 3B) and in their steatotic degeneration (Figure 3D), as well as a prominent decrease in the number of hepatic lipid droplets positive to oil red O staining (Figure 3F). When compared to those of the NASH group, the NASH + 100 mg/kg γ-MCA group exhibited significant reduction in liver index and non-significant difference in body weight (Figure S1). The significant downregulation of the steatosis score (NASH vs. NASH + 100 mg/kg γ-MCA: 2.7500 ± 0.1637 vs. 0.6250 ± 0.1830, *p* = 0.012) (Figure 3C) and hepatic TG content (NASH vs. NASH + 100 mg/kg γ-MCA: 0.8556 ± 0.0630 mmol/g vs. 0.4186 ± 0.0765 mmol/g, *p* = 0.0013) (Figure 3E) indicated an anti-steatosis role of γ-MCA in the rodent NASH. Being consistent with its pharmacological activity in vitro, γ-MCA showed the best therapeutic effect at a dosage of 100 mg/kg rather than 10 mg/kg (Figure 3B–F).

### 3.3. Steatotic Amelioration Reduced Hepatic Lipid Peroxidation

**Figure 4:**
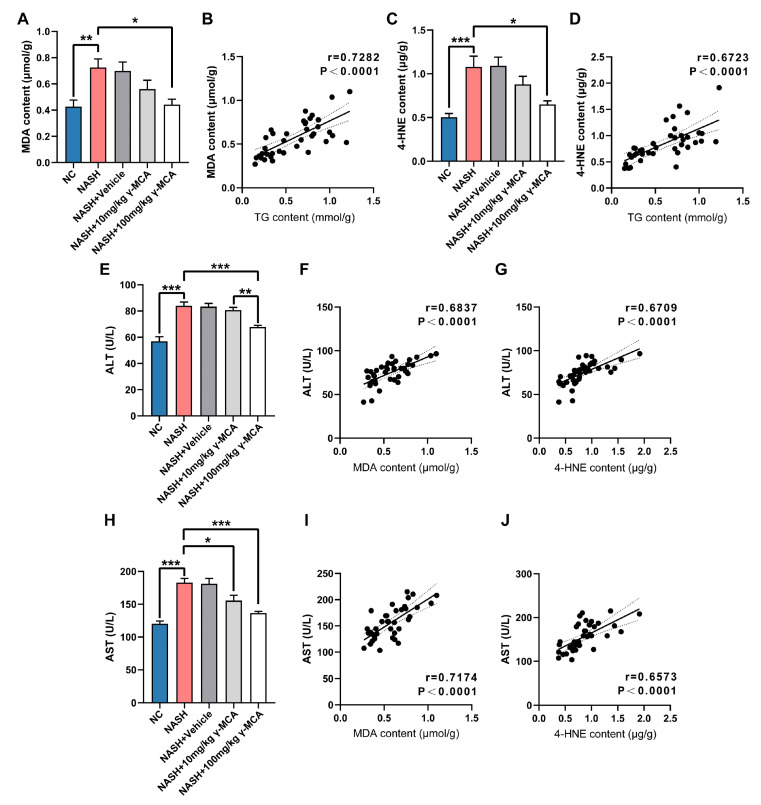
γ-MCA-based steatotic attenuation mitigated lipid peroxidation and oxidative impairment. (**A**–**D**) Both MDA (**A**) and 4-HNE (**C**) were upregulated in the NAFLD group and significantly reduced in the 100 mg/kg γ-MCA group, with strong correlations with hepatic TG content (**B**,**D**). (**E**–**J**) The NAFLD group exhibited an increase in the serum levels of ALT (**E**) and AST (**H**), both of which experienced a significant decrease in the 10 mg/kg γ-MCA and/or NAFLD + 100 mg/kg γ-MCA groups. In addition, both ALT and AST were positively associated with MDA (**F**,**I**) and 4-HNE (**G**,**J**). The data were presented as the mean ± SEM. *, *p* < 0.05; **, *p* < 0.01; and ***, *p* < 0.001.

The products of lipid peroxidation, MDA and 4-HNE, were analyzed to highlight the impact of γ-MCA-based steatotic attenuation. In the NASH group, hepatic levels of both peroxides underwent a significant increase in comparison to those of the NC group (Figure 4A,C). On the contrary, γ-MCA treatment dose-dependently reduced the production of lipid peroxides. As compared to their ascending levels in the NASH group, MDA (NASH vs. NASH + 100 mg/kg γ-MCA: 0.7255 ± 0.0645 μmol/g vs. 0.4419 ± 0.0412 μmol/g, *p* = 0.0141) and 4-HNE (NASH vs. NASH + 100 mg/kg γ-MCA: 1.0780 ± 0.1249 μg/g vs. 0.6499 ± 0.0405 μg/g, *p* = 0.0104) were downregulated with statistical significance after 100 mg/kg γ-MCA gavage (Figure 4A,C). The positive correlation of TG content and lipid peroxides, with related coefficients 0.7282 and 0.6723, both of which are *p* < 0.0001 (Figure 4B,D) convinced the causality between steatotic attenuation and peroxidative inhibition.

### 3.4. Mitigation of Peroxidative Injury and Apoptosis Assisted Prevention of NASH

In our study, mice with liver steatosis and related lipid peroxidation (NASH group) suffered from a significant increase in serum transferases (ALT, AST) (Figure 4E,H). These indexes of hepatocyte injury experienced prominent normalization in the NASH + 10 mg/kg γ-MCA and/or NASH + 100 mg/kg γ-MCA groups (Figure 4E,H), mimicking those of the NC group. The elevation of ALT and AST levels also took place upon the excessive production of MDA (Figure 4F,I) and 4-HNE (Figure 4G,J).

**Figure 5:**
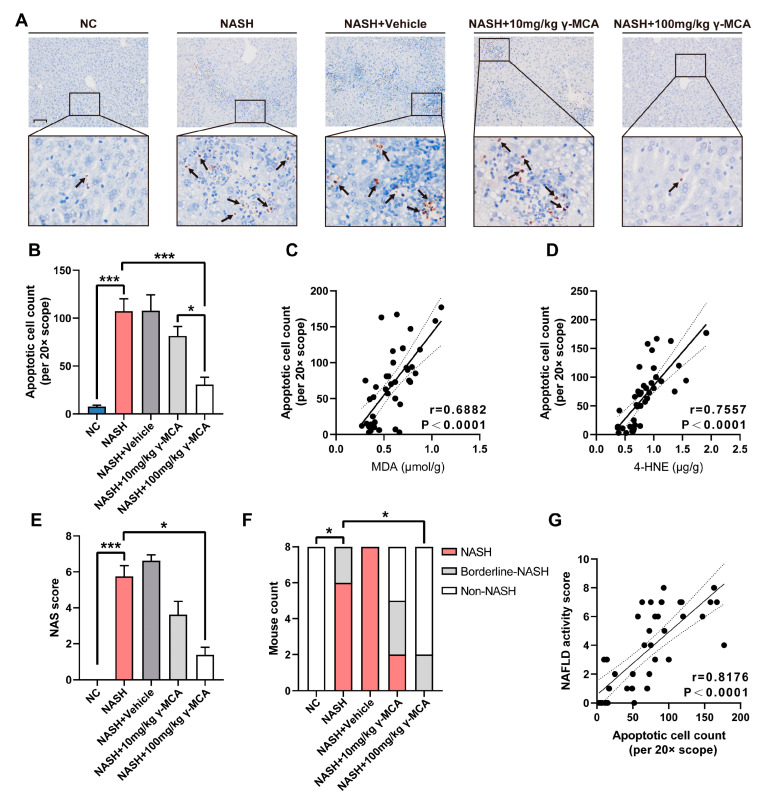
Relief of lipid peroxidative injury ameliorated hepatocyte apoptosis and NASH. (**A**) A TUNEL assay displayed plentiful apoptotic hepatocytes in lobular inflammatory foci of the NAFLD group, but rare apoptosis was documented in both NC and 100 mg/kg γ-MCA groups (scale bar: 50 μm). Arrows highlighted the apoptotic hepatocytes. (**B**) In contrast to high-level hepatocytic apoptosis in the NAFLD, 100 mg/kg γ-MCA treatment exerted an effect of apoptotic protection with statistical significance. (**C**,**D**) The apoptotic cell count demonstrated a positive correlation with both MDA (**C**) and 4-HNE content (**D**). (**E**,**F**) When compared to those of the NAFLD group, 100 mg/kg γ-MCA treatment led to a significant reduction in the NAS score (**E**) and the mouse count with NASH (**F**). (**G**) The Spearman correlation analysis showed a close correlation between the apoptotic cell count and the NAFLD activity score. The data were presented as the mean ± SEM. *, *p* < 0.05; ***, *p* < 0.001.

By a TUNEL assay, peroxidative injury in the NASH group led to multiple hepatocyte apoptosis near the inflammatory foci, which was rarely detected in the NC group (NC vs. NASH: 7.6250 ± 1.4873 vs. 107.2500 ± 13.0750, *p* < 0.0001) (Figure 5A,B). However, γ-MCA treatment dose-dependently prevented hepatocytes from the apoptotic process (NASH vs. NASH + 100 mg/kg γ-MCA: 107.2500 ± 13.0750 vs. 30.6250 ± 7.6763, *p* = 0.0002; NASH + 10 mg/kg γ-MCA vs. NASH + 100 mg/kg γ-MCA: 81.3750 ± 9.9641 vs. 30.6250 ± 7.6763, *p* = 0.0198) (Figure 5A,B). The apparent association between peroxides and the count of apoptotic hepatocytes presented a key part of lipid peroxidation in injury-based apoptosis (Figure 5C,D).

Hepatocyte apoptosis serves as an important trigger of lobular inflammation and, together with hepatocyte steatosis and ballooning, ultimately introduces NASH. Thus, a high incidence of NASH (6/8) and borderline-NASH (2/8) occurred in the NASH group (Figure 5E,F). With the mitigation of hepatocyte apoptosis, 100 mg/kg γ-MCA effectively protected mice from NASH (NASH, 0/8; borderline-NASH, 2/8) by the decrease in the NAS score (NASH vs. NASH + 100 mg/kg γ-MCA: 5.7500 ± 0.5900 vs. 1.3750 ± 0.4199, *p* = 0.0424) (Figure 5E,F). The close correlation of apoptotic cell count and NAS score provided further evidence for mechanisms of γ-MCA treatment (Figure 5G), mainly on a basis of improvement in peroxidative injury and apoptosis.

### 3.5. FXR-Based Inactivation of Lipogenesis Characterized γ-MCA Administration In Vivo

**Figure 6:**
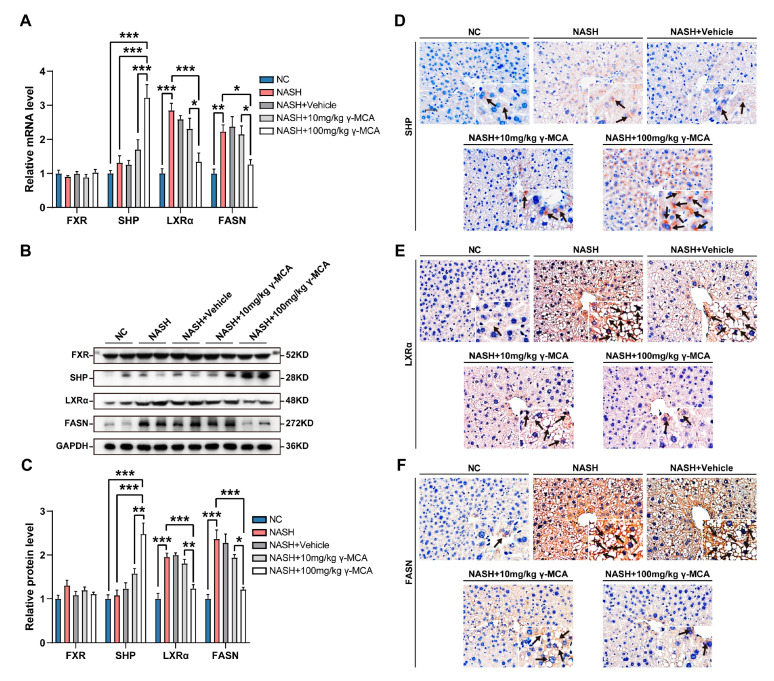
γ-MCA activated FXR/SHP/LXRα/FASN signaling in vivo. (**A**) The SHP was transcriptionally activated by the 100 mg/kg γ-MCA-based activation of FXR, and then inhibited the HFHC diet-induced expression of LXRα and FASN. (**B**,**C**) Western blot (**B**) and semi-quantitative analysis (**C**) revealed that SHP was upregulated upon the 100 mg/kg γ-MCA treatment, accompanied by the downregulation of LXRα and FASN expression. (**D**–**F**) Immunohistochemical staining verified the induction of SHP (**D**) and the expressive inhibition of both LXRα (**E**) and FASN (**F**) in hepatocytes after 100 mg/kg γ-MCA treatment (scale bar: 25 μm). Arrows indicate the positive parts of hepatocytes. The data were presented as the mean ± SEM. *, *p* < 0.05; **, *p* < 0.01; and ***, *p* < 0.001.

In the NASH group without γ-MCA exposure, there was hepatic LXRα and FASN expression much higher than those of the NC group (Figure 6A–D). The levels of FXR and SHP were kept constant (Figure 6A–D). Contrastively, the agonist effect of γ-MCA on FXR-activated SHP expression at both transcriptional (NASH vs. NASH + 100 mg/kg γ-MCA, *p* < 0.0001) and translational levels (NASH vs. NASH + 100 mg/kg γ-MCA, *p* = 0.0002) (Figure 6A–C). The upregulated SHP exerted an inhibitory impact on LXRα and, successively, on FASN expression (NASH vs. NASH + 100 mg/kg γ-MCA, *p* = 0.0213 in the transcriptional level, *p* = 0.0003 in the translational level; NASH + 10 mg/kg γ-MCA vs. NASH + 100 mg/kg γ-MCA, *p* = 0.0410 in the transcriptional level, *p* = 0.0168 in the translational level) (Figure 6A–C). Immunohistochemical analysis showed that at high level of SHP and low levels of LXRα, FASN were more represented among the hepatocytes in the NASH + 100 mg/kg γ-MCA group than in the NASH group (Figure 6D–F). The semiquantitative analyses were performed to precisely describe the expressive differences of SHP, LXRα, and FASN between various groups (Figure S2). Finally, evident downregulation of FASN underlays lipogenic inactivation and steatotic attenuation in hepatocytes of γ-MCA-treated mice. 

## 4. Discussion

FXR, an important member of the nuclear receptor superfamily, has been well described as having a crucial role in hepatic lipid metabolism [6]. Although the FXR signaling may not change significantly between NASH patients and healthy controls, FXR has been considered as a potential therapeutic target of NASH. Upon activation, FXR induces the expression of SHP by targeting the inverted repeat separated by one nucleotide (IR-1) FXR response element [19]. Then, SHP dimerizes with LXRα to abrogate its effect on metabolic genes (e.g., FASN) [20,21,22,23]. In contrast to the LXRα-dependent transcriptional activation of FASN, through the direct repeats separated by four nucleotides (DR-4) of the LXR response element it predominantly downregulates the condition of SHP-based LXRα inactivation [24,25]. The major action of FASN, namely de novo lipogenesis, is ultimately inhibited in hepatocytes. In our study, high-dose γ-MCA treatment significantly increased the hepatic SHP levels in vivo and in vitro. An antagonist effect of SHP on LXRα repressed the FASN expression, which resulted in the descendent concentration of hepatocellular TG. On the contrary, FXR knockdown abolished the pharmacological effect of γ-MCA on SHP and successively abolished LXRα at both transcriptional and translational levels. γ-MCA-induced TG reduction was also absent in these FXR-lacking hepatocytes. Therefore, γ-MCA functions to inhibit lipogenesis and related hepatic steatosis via the FXR/SHP/LXRα/FASN signaling.

Hepatic steatosis represents the hallmark of NASH, mainly by means of peroxidative impairment. Mechanically, lipid overload disturbs the electron transport chain (ETC) (e.g., complex I and III) and enhances the fatty acid oxidation (FAO) [26], while a large generation of reactive oxygen species (ROS) is attributed to the FAO-related enzymes (e.g., long chain acyl-CoA dehydrogenases and very long-chain acyl-CoA dehydrogenases) [27,28]. As a result, increased production and outflow of ROS take place in mitochondria. The reaction of ROS and fatty acid produces toxic peroxides, such as MDA and 4-HNE [29]. Contrastively, γ-MCA-induced steatotic attenuation demonstrated a correlation with reduced hepatic peroxides (MDA and 4-HNE) in the present experiments.

Among these lipid peroxides, MDA has been shown to modify multiple proteins in an MDA-acetaldehyde (MAA) modification manner and injure hepatocytes by disrupting the membrane integrity. Similar impairments occur in hepatocytes after their exposure to 4-HNE [30,31,32]. When compared to the peroxide-related transferase (ALT and AST) upregulation in the NASH group, these indexes of hepatocyte injury underwent improvement in both NASH + 10 mg/kg γ-MCA and/or NASH + 100 mg/kg γ-MCA groups. Taking into account its inhibitory action in lipid peroxidation, γ-MCA was convinced to exert a protective impact against peroxidative injury. Injurious stimuli, especially steatosis-based lipid peroxidation, bring about apoptosis of hepatocytes in NASH [33,34]. First, peroxidative abrogation of lysosomal integrity initiates caspase-3-dependent apoptosis [35]. Second, the MAA-modified proteins exhibit proapoptotic characteristics upon MDA exposure [36,37]. Moreover, 4-HNE serves as a universal activator of apoptosis by multiple methods, including the activation of calpain, caspase-3 [38], p53 [39,40,41], and Fas-mediated ASK1/JNK/Caspase-3 signaling [41,42]. By the γ-MCA-dependent alleviation of peroxidative impairment, hepatic apoptosis was greatly reduced in the NASH + 100 mg/kg γ-MCA group as compared to that of the NASH group.

Apoptosis of hepatocytes results in chemokine-related inflammation through the activation of caspase-3 and nuclear translocation of activator protein-1 [43]. The activated Fas also promotes an inflammatory response by caspase-8-dependent activation of NLRP3 inflammasome and maturation of IL-1β [44]. BAX/BAK signaling for apoptosis triggers inflammation by both caspase-8 and NLRP3-induced IL-1β secretion [45]. In addition, hepatocytes in mice with a high-fat diet release high-mobility group box1 (HMGB1), which activates Kupffer cells to stimulate inflammation [46]. Consistently, exacerbated hepatic apoptosis features NASH patients, with a positive correlation with inflammatory activity [47]. By its anti-apoptotic property, pan-caspase inhibitor (VX-166) contrastively reduces hepatic inflammation, serum ALT levels, and liver fibrosis [48,49]. Taken together, apoptosis-based inflammation underlies NASH. Dramatically, high-dose γ-MCA treatment (100 mg/kg) in our experiments prevented hepatic apoptosis to mitigate lobular inflammation in mice. The resultant reduction of the NAS score confirmed the therapeutic effect of γ-MCA against NASH.

Strong agonists of FXR (e.g., obeticholic acid (OCA)) were synthesized for the NASH treatment. However, intensive activation of FXR gives rise to upregulated low-density lipoprotein (LDL)-cholesterol and downregulated high-density lipoprotein (HDL)- cholesterol. This dysmetabolism of lipoproteins is attributed to the generation of LDL by apoC II and apoC III imbalance [50,51,52] and the increased clearance of HDL by the hepatocellular expression of CETP and the scavenger receptor-B1 [53,54]. Moreover, The FXR-based upregulation of lithocholic acid (LCA), the strongest agonist of TGR5, may contribute to TGR5-induced pruritus, which was a concern in the OCA clinical trial [55,56,57,58,59]. Thus, a moderate but not strong agonist of FXR is suggested for the strategy of NASH therapy. γ-MCA, with its natural, moderate activity as an FXR agonist indeed exerts therapeutic action against NASH.

There are some limitations in this study. First, we focus our experiments on NAFLD with hepatic TG accumulation, though investigation of serum TG and TC could highlight the effect of γ-MCA on other metabolic disorders associated with NAFLD. Mice exposed to γ-MCA did not exhibit abnormality in dietary intake, behavior, appearance, and faeces, but further analyses will be valuable to take deep insight into the possibility of adverse effects and the long-term outcomes.

## 5. Conclusions

**Figure 7:**
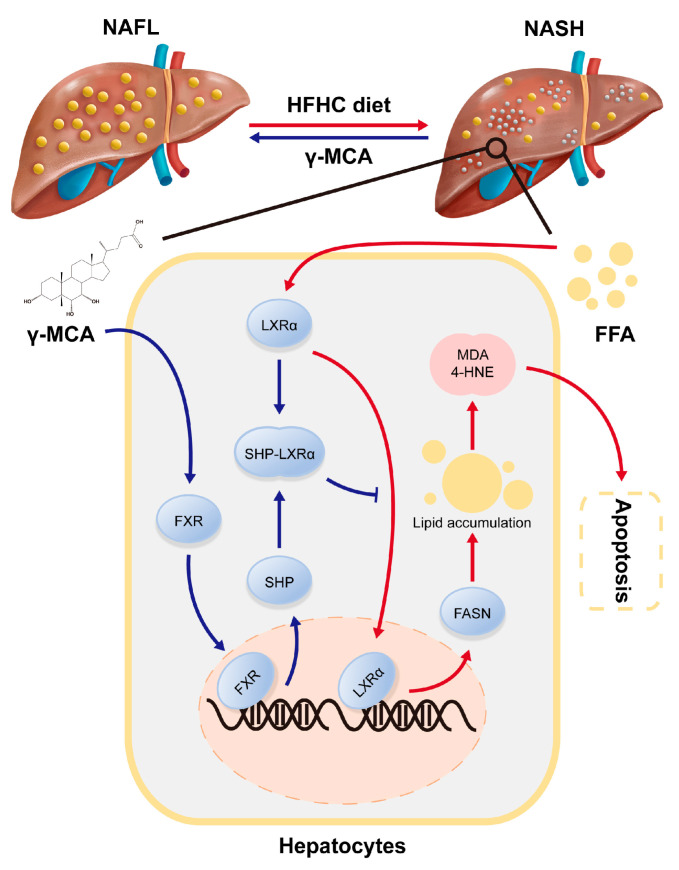
Schematic model depicting the inhibitory effect of γ-MCA on NASH via FXR/SHP/LXRα/FASN signaling. The γ-MCA-activated FXR upregulates the expression of SHP, which dimerizes LXRα to transcriptionally inhibit its target genes (e.g., FASN). Inhibition of FASN reduces de novo lipogenesis in response to FFA, and then abolishes the lipid accumulation in hepatocytes. Steatotic attenuation leads to decreased lipid peroxidative products (e.g., MDA, 4-HNE) and the successful diminution of hepatocytic apoptosis. The apoptosis-dependent lobular inflammation is finally abrogated to prevent progress from NAFL to NASH.

NASH serves in the key step from NAFL to hepatic fibrosis/cirrhosis and HCC depending on steatosis-based peroxidative impairment. The present study uncovers that γ-MCA agonizes FXR to inhibit the lipogenesis of hepatocytes via SHP/LXRα/FASN signaling. The lipogenic inactivation attenuates liver steatosis induced by an HFHC diet. An amelioration of hepatic steatosis protects hepatocytes from lipid peroxidation and related injury. The abolishment of injurious apoptosis finally prevents NASH in the rodent model (Figure 7). These findings highlight an inhibitory effect of γ-MCA against steatosis-induced peroxidative injury, and the mitigation of NASH by targeting FXR/SHP/LXRα/FASN signaling. It suggests a potential strategy for NASH treatment by γ-MCA.

## Figures and Tables

**Table 1:** Specific primers for RT-QPCR.

Name (Symbol, ID)	Type	Sequence (5′-3′)	Product Size
FXR (Nr1h4, 20186)	Forward	GCAACCAGTCATGTACAGATTC	143 bp
	Reverse	TTATTGAAAATCTCCGCCGAAC	
SHP (Nr0b2, 23957)	Forward	GTCCGACTATTCTGTATGCACT	162 bp
	Reverse	CTACTGTCTTGGCTAGGACATC	
LXRα (Nr1h3, 22259)	Forward	GAGTGTCGACTTCGCAAATG	87 bp
	Reverse	CTTCAGTTTCTTCAAGCGGATC	
FASN (Fasn, 14104)	Forward	TAAAGCATGACCTCGTGATGAA	230 bp
	Reverse	GAAGTTCAGTGAGGCGTAGTAG	
GAPDH (Gapdh, 14433)	Forward	GGTTGTCTCCTGCGACTTCA	183 bp
	Reverse	TGGTCCAGGGTTTCTTACTCC	

## Data Availability

The datasets created and analyzed in the current study can be found in the Jianguoyun cloud service at https://www.jianguoyun.com/p/DYlImSEQt6i2ChiituwEIAA.

## Supplementary Materials

The following supporting information can be downloaded at: https://www.mdpi.com/article/10.3390/nu15051255/s1, Figure S1: Effects of γ-MCA on body weight and liver weight; Figure S2: Semiquantitative analyses of immunohistochemical signals.
