# Supplementary material for: Gamma-Muricholic Acid Inhibits Nonalcoholic Steatohepatitis: Abolishment of Steatosis-Dependent Peroxidative Impairment by FXR/SHP/LXRα/FASN Signaling
Source: Nutrients. 2023 Mar 2;15(5):1255. doi: 10.3390/nu15051255 (PMC10005659; doi:10.3390/nu15051255)
Supplement: Supplementary file 1 [file nutrients-15-01255-s001.zip › nutrients-2212134-supplementary.pdf]

Supplementary Materials

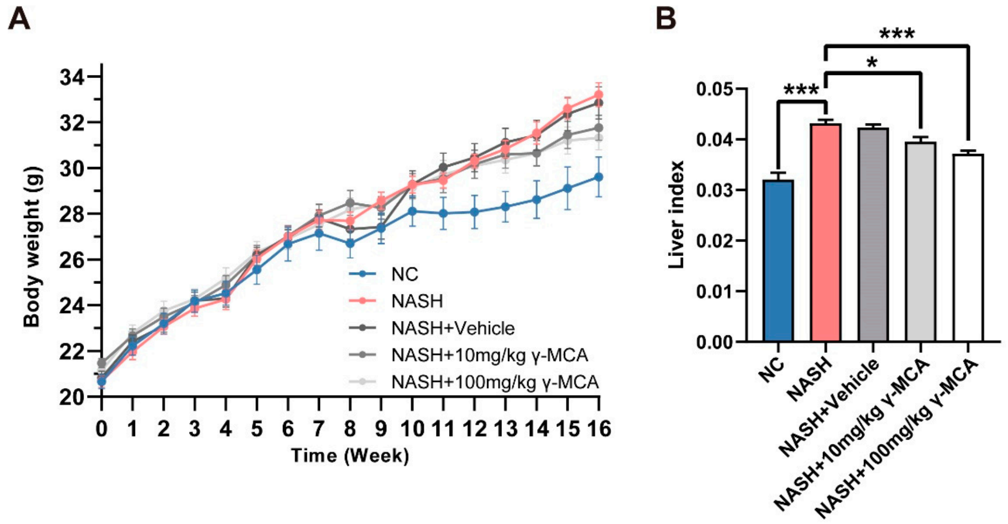

Figure S1. Effect of γ-MCA on body weight and liver weight. (A-B) The alterations of body weight (A) and liver index (B) during HFHC diet and/or γ-MCA exposure. The data were presented as mean ± SEM. *, P<0.05; ***, P<0.001.

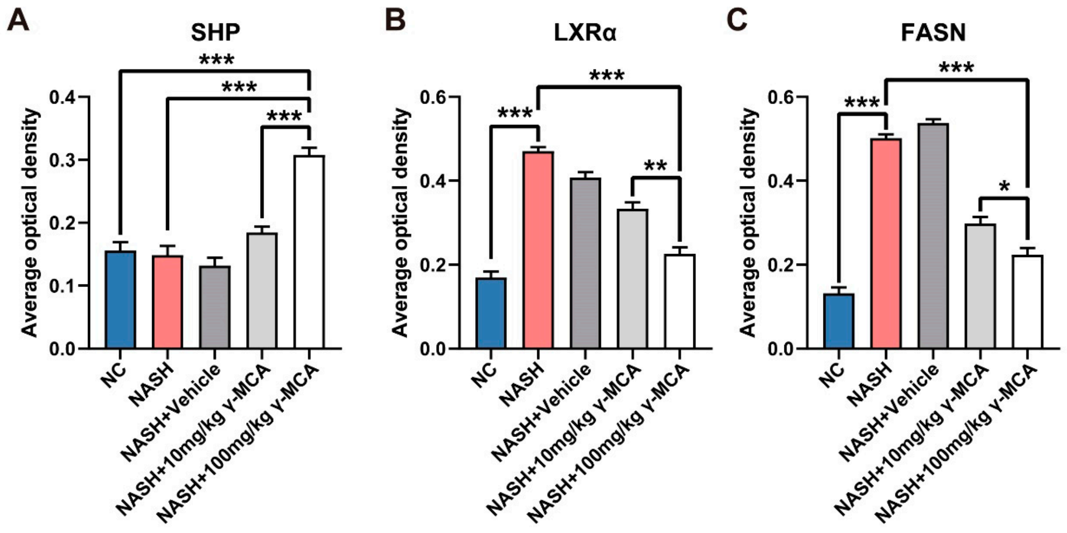

Figure S2. Semiquantitative analyses of immunohistochemical signals. The average optical density showed the significant upregulation of SHP (A), and then downregulation of LXRα (B) and FASN (C), in the NASH+100mg/kg γ-MCA group, compared with the NASH group. The data were presented as mean ± SEM. *, P<0.05; **, P<0.01; ***, P<0.001.
